# 10 years of PET/MR: Looking back for a moment

**DOI:** 10.1007/s12350-020-02358-z

**Published:** 2020-09-09

**Authors:** Stephan G. Nekolla

**Affiliations:** 1 School of Medicine, Department of Nuclear Medicine, Technische Universität München, Munich, Germany; 2 DZHK (Deutsches Zentrum für Herz-Kreislauf-Forschung e.V.), Partner Site Munich Heart Alliance, Munich, Germany

Time flies and this holds true even in these remarkable days. Almost 10 years ago, the first fully integrated clinical PET/MR capable of truly simultaneous measurements was installed in our department. I would like to use this opportunity and reflect on a few (and really not all) things which preceded it and what I experienced: on the introduction of the PET/CT, the concepts we envisioned beforehand, the lessons we learned, and what a summary could be after almost a decade working with one of the most complex and most expensive systems in non-invasive medical imaging. It goes without saying that this is a very personal perspective.

Introduced with substantial marketing fanfare and quite some engineering efforts, the “beast” as I used to call it went from a well-kept secret to an amazing technical achievement made available to the medical imaging community. Having this said, let me rapidly limit the scope: the hardware was truly amazing but the “software” was not quite ready. The hyphens indicate that not only the computer software running on the “beast” had its issues but also the “software” of the team running it.

In principle, we were very well prepared. I had the privilege to join Markus Schwaiger’s group in 1993: back then, we were able to establish rather rapidly one of the very early centers in Europe running a clinical PET service—and gratefully receiving considerable support from colleagues based all over the world. The integration of sequentially acquired cardiac PET and cardiac MRI was actually a focus starting very early; the validation of MRI’s capabilities to assess perfusion^1^ and viability^2^ was actually completed just before the clinical introduction of the first hybrid imaging systems, SPECT/CT and PET/CT. This transition to hybrid systems was actually a watershed moment: it ended the dominance of “single bed position” procedures scanning the heart or the brain (both fitted well into the PET scanners axial field of view and allowed routinely dynamic or gated acquisitions) (Figure 1). Oncological imaging was back then rather limited due to time constraints. This changed dramatically with the PET/CT and the simple fact that the CT scan replaced the time-consuming transmission scan—basically the *conditio sine qua non* to generate attenuation corrected and thus quantitative images. The increase in scanner sensitivity by going from 2D to 3D acquisitions and increasingly powerful scanner hardware enabled whole-body scan times in the order of 15 to 20 minutes. This introduced quite a challenge for the “non-onco” imaging folks: whereas it was not that complicated from a logistical perspective to get a scanning slot for a heart or a brain study before, now we had to justify that we “blocked” three whole-body scans with a single heart scan. Consequentially, the—in the good old days—exotic oncological scan put cardiac and neuro-imagers now in precisely *that* position: almost bizarre. Quite ironically, a sentence such as “due to the increasing availability of PET/CT systems for oncological applications those systems offer also potential for cardiac applications” can be found in more than one review article on the advantages of cardiac PET/CT.^3^,^4^ Thus, the upcoming availability of PET/MR initially created quite some optimism that new scan time (assuming naively that this translated into new applied research time) was around the corner. Unfortunately, this was not necessarily true. Several factors contributed to this. The first obstacle was attenuation correction, which centers directly on the corner stone of PET: reliable quantification. We suggested quite early a robust approach^5^ and our community put an amazing effort into the investigation of a multitude of techniques to improve this into the field. Unfortunately, it created also quite some reservations within the clinical users^1^. In particular, we observed in a long series of software upgrades that the algorithms changed and the quantification—although normally on a quite small level—changed as well. But in general, as reproducibility especially in the context of any therapy response monitoring is a hallmark of PET, variability is simply not desirable. We recently re-evaluated after one decade the attenuation correction models including a sophisticated bone model and came to the conclusion that we actually did pretty well back then.^6^ But nevertheless, if confidence in something basic such as attenuation correction was lacking, quite some users of standard or even advanced clinical application were somewhat puzzled.

**Figure 1:**
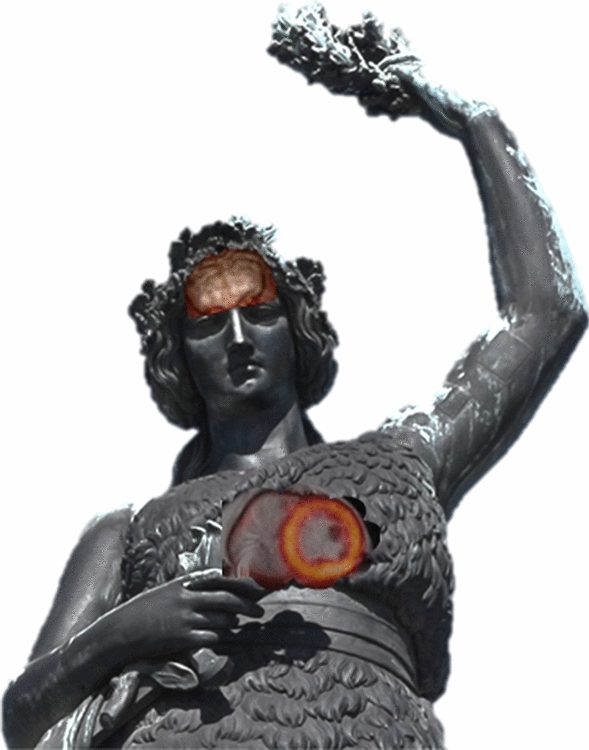
The Bavarian “guardian angel” (“Patronae Bavaria”)—sitting above the Oktoberfest (canceled this year for well-known reasons). The author took the liberty to reveal brain and cardiac structures based on PET/MRI data

There is another factor quite basic which was initially not fully realized but surfaced rather rapidly with patients: the PET detector ring reduces the diameter of a 70 cm bore to only 60 cm. Although many MRI systems still feature such a diameter, the added “comfort” of 70 cm systems (introduced quite some years ago) was instantaneously missed when the PET/MR went into operation. This has certainly a profound effect on patients with cardiovascular disease with still rather long examinations times—and an elevated BMI does not necessarily help here.

The other observation over these years was not technical or logistical but had overall a large effect. The PET/CT integrated two imaging modalities with a rather modest feature set (typically whole-body PET and either low-dose or diagnostic CT), required only a modest amount of training, and the PET and CT images were so different in features and appearance that they got along with each other almost perfectly. This aspect of integration was different for PET and MRI . The dissimilarities happened on different levels: MRI is a much more “real-time” modality where adjustments to imaging sequences are—at least for the applications I have in mind—not rare. In other words, there is a level of interactivity, which is not necessarily the case both for PET and CT. This interactivity might arise from patient-specific artifacts which need sequence parameter adjustments or simply from complex sequences where the default settings simply do not work. This requires a well-trained, interdisciplinary team which provides the necessary level of flexibility during the acquisition. That the number of acquired images is well beyond that of a PET/CT scan (not to mention registration between the PET and all the MRI sequences) makes things not easier: Beyond this more logistical issue lingers, however, a more serious threat: I actually did my Ph.D. many years ago in MRI and until I started in Munich, I was not aware that a nuclear medicine department actually existed. We grew up simply under the impression that MRI is the number one imaging modality. End of story.

In other words (let me exaggerate here), from the point of view of dyed-in-the-wool MRI folks, there is no need for another modality providing information with radioactive (!) molecular imaging. This certainly does in no way reflect the opinion of PET/MRI users—but I am afraid that the latter are outnumbered. In fact, I ran more than once into puzzled faces when asking colleagues from the “pure teaching” about collaborative projects—and this holds also true for the industry. Business units which used to more or less compete for customers needed now to cooperate—potentially jeopardizing their own territory.

A dominating factor making life with the “beast” quite troublesome are the initial and the running costs. Not unexpectedly (as PET and MRI per se are rather expensive modalities) the fully integrated systems belonged and still belongs to the most expensive systems for (potentially routine capable) non-invasive, medical imaging. Only recently, the total-body PET systems were able to top this. In principle this should be no show stopper: but unfortunately only a few of us operate in dedicated research environments where so mundane issues such as short- and medium-term cost-effectiveness do not matter. For all the others, a simple calculation could be done: a system with twice the price of a PET or a MRI but only half (or less) the throughput of a PET/CT is in trouble. This is not simplified by the annual maintenance costs of about 5% to 10% of the initial system price. Thus, the continuing financial load is a factor which should not be underestimated. In order to address the throughput, the community was fast to realize that whole or partial body PET scans are not easy to implement and imaging scenarios with a short field of view of about 10 cm—ironically very much like in the early days of PET—are optimal, leading to an increased interest for cardiovascular applications. But even cardiac MRI scans are not particularly fast and “suffer” from a high degree of interaction. Nevertheless, we see a substantial interest for imaging inflammatory diseases—interestingly, should our diagnosis result in implantable devices, it is unlikely that we see this patient again in an MR or PET/MR due to potential incompatibility.

So… PET/MRI is a great research tool and worth any costs? Absolutely, and I wholeheartedly agree—but only to the first point and many researchers really showed impressive results. However, in today’s competitive landscape—and the healthcare sector is no exception—the complete picture counts. Whether a more modestly priced PET/MR with lower magnetic field strength and a smaller field of view—why not in the price range of a digital PET/CT—would be more fitting in the current scenario is plain speculation…but I would think so. Whether cardiovascular imaging is the key target is at least unclear. In our setting, an organ-based (i.e., small field of view) approach, co-driven by the fact that novel radiopharmaceuticals are easier to implement in specific oncological setups, might quite well work (Figure 2 was adjusted accordingly) .

**Figure 2:**
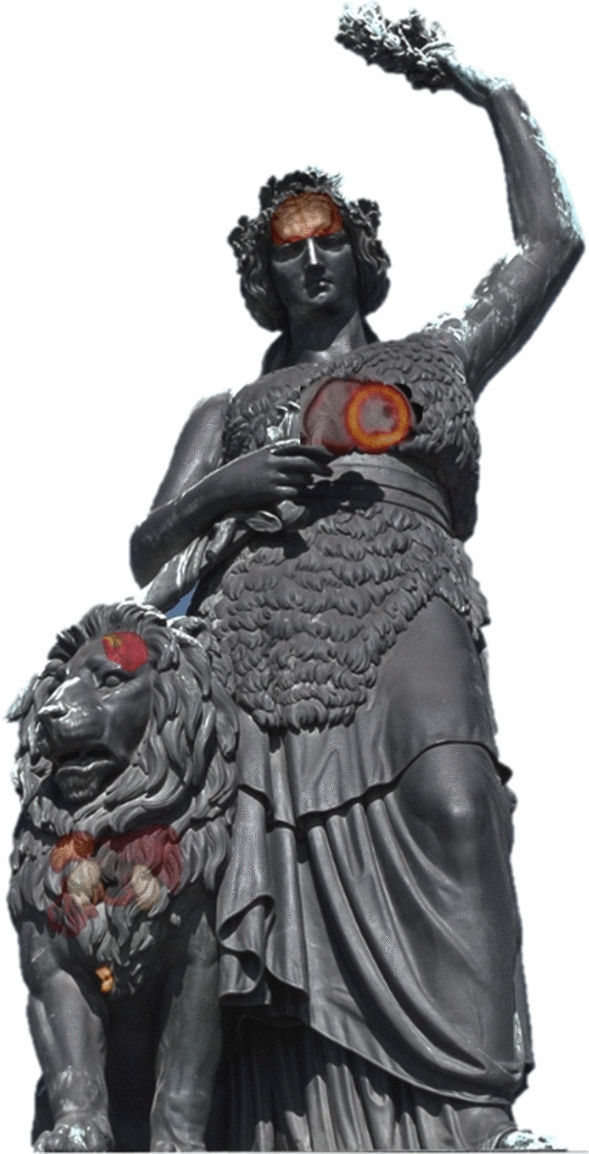
“Bavaria” and her companion adjusted for very particular changes seen in the last decade of PET/MRI

Although it really hurts that our initial enthusiasm was not sustainable, I would not—by any means—miss the last decade: it is always fun riding a “beast.” More seriously, we saw unprecedented means of non-invasive tissue characterization and also several “collateral improvements” which will have substantial impact for future PET imaging: The “digital PET” detectors (created as conventional PMTs are not working in magnetic fields) showed their value in PET/MR and enabled the most recent generation of PET/CT devices. Furthermore, we revisited several projects from the last three decades and learned to appreciate the elegance of mono-modal approaches—myocardial perfusion^7^ and FDG imaging^8^ comes to mind.

But most importantly to me, a new and challenging technology attracted many young research-minded colleagues into our field. This alone was worth it.

## Footnotes

1 It is quite interesting that such an interest was not really detectable for the migration from PET to PET/CT
